# TRAF3 Positively Regulates Host Innate Immune Resistance to Influenza A Virus Infection

**DOI:** 10.3389/fcimb.2022.839625

**Published:** 2022-04-27

**Authors:** Fangzhao Chen, Liurong Chen, Yinyan Li, Huiting Sang, Chunyu Zhang, Shuofeng Yuan, Jie Yang

**Affiliations:** ^1^National Medical Products Administration (NMPA) Key Laboratory for Research and Evaluation of Drug Metabolism, Guangdong Provincial Key Laboratory of New Drug Screening, School of Pharmaceutical Sciences, Southern Medical University, Guangzhou, China; ^2^Department of Pharmacy, Shenzhen Children’s Hospital, Shenzhen, China; ^3^Department of Microbiology, Li Ka Shing Faculty of Medicine, The University of Hong Kong, Hong Kong, Hong Kong SAR, China; ^4^State Key Laboratory of Emerging Infectious Diseases, Li Ka Shing Faculty of Medicine, The University of Hong Kong, Hong Kong, Hong Kong SAR, China

**Keywords:** TRAF3, influenza A virus, antiviral immunity, type-I IFNs, structural domain

## Abstract

Tumor necrosis factor receptor-associated factor 3 (TRAF3) is one of the intracellular adaptor proteins for the innate immune response, which is involved in signaling regulation in various cellular processes, including the immune responses defending against invading pathogens. However, the defense mechanism of TRAF3 against influenza virus infection remains elusive. In this study, we found that TRAF3 could positively regulate innate antiviral response. Overexpression of TRAF3 significantly enhanced virus-induced IRF3 activation, IFN-β production, and antiviral response, while TRAF3 knockdown promoted influenza A virus replication. Moreover, we clarified that inhibiting ubiquitinated degradation of TRAF3 was associated with anti-influenza effect, thereby facilitating antiviral immunity upon influenza A virus infection. We further demonstrated the key domains of TRAF3 involved in anti-influenza effect. Taken together, these results suggested that TRAF3 performs a vital role in host defense against influenza A virus infection by the type-I IFN signaling pathway. Our findings provide insights into the development of drugs to prevent TRAF3 degradation, which could be a novel therapeutic approach for treatment of influenza A virus infection.

## Introduction

Influenza viruses, especially influenza A virus (IAV), can cause global pandemics, posing serious threat to public health and economic development. Infection with highly pathogenic IAV may cause acute respiratory distress syndrome (ARDS) and even death in humans (Herold et al., 2015). As reported, mutations of key amino acid sites on influenza hemagglutinin (HA) can cause the cross-species transmission of influenza virus, laying hidden risks for the spread of influenza, a zoonotic disease, to human society (Herfst et al., 2012). Due to the rapid mutation rate of IAV antigen and the ongoing challenge of resistance to the existing antiviral drugs (Weinstock and Zuccotti, 2006; Choi et al., 2018), it is in urgent need of diverse strategies for prevention and treatment of IAV infection.

The innate immune system, which provides the first line of host defense against the invasion of exogenous pathogens, will initiate a wide variety of signaling pathways, ultimately leading to the secretion of inflammatory cytokines (Chen et al., 2018). Innate immunity is also critical in the initial containment of IAV infection. During IAV infection, the host pathogen recognition receptors (PPRs) can recognize viral conserved components called pathogen-associated molecular patterns (PAMPs), leading to the activation of innate immune signaling that finally induces the production of antiviral cytokines like interferons (IFNs), which could prevent virus replication and spread (Schneider et al., 2014; Cao, 2016; Chen et al., 2018). With the characteristics of broad spectrum, rapid response, and less drug resistance, host factors in response to viral infection have become a treasure trove of targets for new antiviral approaches. Several host genes have been reported to be associated with the virus pathogenesis and host immune response, such as TRIM25 (tripartite motif-containing protein 25) (Gack et al., 2009) and PAPR1 (poly(ADP-ribose) polymerase 1) (Xia et al., 2020), bringing a promising prospect for the search of potential anti-influenza virus targets from the innate immune signaling pathways.

TRAF3 (tumor necrosis factor receptor-associated factor 3), a member of the tumor necrosis factor receptor-associated factor (TRAF) family, plays a significant role in the regulation of inflammation and antiviral immunity (Guven-Maiorov et al., 2016; Bishop et al., 2018). The longest isoform of TRAF3 encoded by the classical transcript has 568 amino acids, which can be divided into a RING finger domain, five zinc finger domains and a TRAF domain containing a α-helical coiled-coil region (TRAF-N), and an eight-stranded anti-parallel β-sandwich structure (TRAF-C, also called MATH domain) (Bradley and Pober, 2001; He et al., 2007). Some domains are important for the biological activity of TRAF3. For instance, protein ubiquitination mediated by the RING finger domain plays a key role in the mechanism of TRAF-dependent signal transduction (Bhoj and Chen, 2009). TRAF-N and TRAF-C domains can interact with downstream factors like cIAP1/cIAP2 or NF-κB-induced kinase (NIK) in the process of receptor activation (Hacker et al., 2011).

Emerging evidence pointed out that TRAF3 might act as an important immunomodulator that contributes to innate immune defense against invading pathogens (Paz et al., 2011; Zhu et al., 2019). TRAF3 is crucial for controlling virus-triggered type I IFN response and thus initiates a series of downstream effects (Oganesyan et al., 2006; Hacker et al., 2011). Recent studies have shown that TRAF3 is critical to activating the host innate immunity against a range of viruses (Oganesyan et al., 2006; Perez et al., 2010; Zhang et al., 2018) and thus inhibiting virus replication. However, the interaction of TRAF3-mediated innate immunity with IAV infection has not been demonstrated clearly.

In this study, we investigated the underlying influences of TRAF3 in the initiation of type I interference (IFN) response to influenza virus infection and clearance. Additionally, we also explored the critical domain of TRAF3 for its immune regulatory function. This research would be helpful for further understanding of the immune function of TRAF3 and the molecular mechanism against IAV infection and provide a theoretical basis for prevention and treatment of IAV.

## Material and Methods

### Cells and Viruses

Madin-Darby canine kidney (MDCK) cells, A549 cells, and 293T cells were purchased from ATCC. MDCK cells and 293T cells were incubated in DMEM medium containing 10% FBS and 1% penicillin/streptomycin solution at 37°C in a humidified atmosphere of 5% CO_2_. A549 cells were cultured in RIPM-1640 medium containing 10% FBS and 1% penicillin/streptomycin solution at 37°C in a humidified atmosphere of 5% CO_2_. The infected cells were cultured in FBS-free medium containing 1 μg/ml TPCK trypsin after contacting the tested virus.

The H1N1 influenza A virus strain (A/WSN/1933) was amplified by 10-day-old chicken embryos and kept at -80°C until use.

### Plasmids

The PEZ-Lv242-TRAF3 lentiviral plasmid and its domain deletion plasmids were commercially constructed by GeneCopoeia, Guangzhou, China. The psPAX2, HA-UB, and VSVG packing plasmids of lentivirus were kept in our laboratory. Plasmids of viral ribonucleoprotein complex (vRNP) subunits including pHW2K-NP, pHW2K-PA, pHW2K-PB1, pHW2KPB2, and pPolI-Fluc (firefly luciferase reporter plasmid) used in the mini-replicon system were kindly presented by Professor Bojian Zheng (University of Hong Kong, HK, China). The IgK-IFN-luc plasmid was bought from Miaolingbio, Wuhan, China. The reference plasmid hRluc-TK was obtained from Promega Corporation (Madison, WI, USA).

### siRNA Transfection

A549 cells were transfected with siRNA (GenePharma, Shanghai, China) which targets TRAF3 or as a negative control (NC) at the final concentration of 20 nM by using the EndoFectin Transfection Reagent (GeneCopoeia, China) according to the manufacturer’s instructions. After 4 h of incubation, the medium was replaced by a fresh medium containing 10% FBS and culturing was continued for 24 h to make the transfected cells for the subsequent experiments. Total mRNA was collected after 24 h of transfection to verify the knockdown efficiency of TRAF3 by quantitative real-time PCR (qRT-PCR). The sequences of siRNA are shown in **Table 1**.

**Table 1:** The sequences of siRNA.

Name	Sequence (5′–3′)
si-TRAF3	Sense: 5′-GUUGUGCAGAGCAGUUAAUTT-3′
	Antisense: 5′-AUUAACUGCUCUGCACAACTT-3′
si-Negative control (NC)	Sense: 5′-UUCUCCGAACGUGUCACGUTT-3′
	Antisense: 5′-ACGUGACACGUUCGGAGAATT-3′

### Construction of a TRAF3-Overexpressing Stable Cell Line

The lentivirus system was utilized to construct the stable expression cell lines. Briefly, 293T cells grown in 6-well plates were co-transfected with 1 μg DNA (including 500 ng TRAF3 lentivirus plasmid or vector control plasmid, 250 ng psPAX2 plasmid, and 250 ng VSVG plasmid) using EndoFectin Transfection Reagent. After transfection for 24 h, the original medium was replaced by a fresh complete DMEM+RIPM1640 medium (1:1). Culturing was continued for 24 h. Lentivirus-containing supernatants were harvested and centrifuged, then stored at -80°C or immediately used.

Next, A549 cells were transduced with the above-prepared lentivirus at a ratio of 1:1 with the complete medium containing polybrene (5 μg/ml). The culture medium was replaced with fresh medium at 12 h post-transfection. TRAF3-overexpressing stable cells were successfully obtained by puromycin (2 μg/ml) selection.

### Quantitative Real-Time PCR

Total RNA was extracted from the cells using an RNA Extraction Kit (Foregene, Chengdu, China). RNA extracts were reverse-transcribed into cDNA with PrimeScript RT Reagent Kit (Takara, Shiga, Japan). qRT-PCR was performed by using SYBR Green PCR Master Mix (Promega, USA) and recorded by LightCycler480 (Roche, Basel, Switzerland). In the end, the relative gene expression was analyzed using the method of 2^-ΔΔCT^. Glyceraldehyde 3-phosphate dehydrogenase (GAPDH) was used as a housekeeping gene for normalization. Primers sequences are listed in **Table 2** and **Table S1** (**Supplementary Material**).

**Table 2:** Primer sequences for qRT-PCR.

Name	Primer sequences
PA-forward	5′-AGAGCCTATGTGGATGGATTCG-3′
PA-reverse	5′-TTGGACCGCTGAGAACAGG-3′
TRAF3-forward	5′-TCTTGAGGAAAGACCTGCGAG-3′
TRAF3-reverse	5′-GCGATCATCGGAACCTGACT-3′
IFN-β-forward	5′-ATGACCAACAAGTGTCTCCTCC-3′
IFN-β-reverse	5′-GGAATCCAAGCAAGTTGTAGCTC-3′
TNF-α-forward	5′-CCCAGGGACCTCTCTCTAATCA-3′
TNF-α-reverse	5′-GCTTGAGGGTTTGCTACAACATG-3′
IL-6-forward	5′-AATAACCACCCCTGACCCAAC-3′
IL-6-reverse	5′-TGCTACATTTGCCGAAGAGC-3′
IL-1β-forward	5′-AAATACCTGTGGCCTTGGGC-3′
IL-1β-reverse	5′-TTTGGGATCTACACTCTCCAGCT-3′
GAPDH-forward	5′-AGGGCAATGCCAGCCCCAGCG-3′
GAPDH-reverse	5′-AGGCGTCGGAGGGCCCCCTC-3′

### Western Blotting

Total protein was lysed using RIPA buffer and normalized. Protein samples were separated by 10% SDS-PAGE and then transferred onto PVDF membranes (Roche, Switzerland). The protein membranes were incubated at 4°C overnight with primary antibodies at the appropriate concentrations, followed by incubation with an anti-mouse/rabbit secondary antibody labeled with horseradish peroxidase (Cell Signaling Technology, Danvers, MA, USA; dilution 1:10,000) for 1 h. The protein bands were visualized using a FluorChem E imaging system (ProteinSimple, San Jose, CA, USA). The primary antibodies were used: anti-PB2 (GeneTex, Irvine, CA, USA; dilution 1:1,000), anti-NP (GeneTex; dilution 1:1,000), anti-TRAF3 (Cell Signaling Technology; dilution 1:1,000), anti-IRF3 (Cell Signaling Technology; dilution 1:1,000), anti-HA-UB (Cell Signaling Technology; dilution 1:1,000), and anti-p-IRF3 (Ser386) (Bioss, Shanghai, China; dilution 1:1,000). GAPDH or β-actin was used as a protein control.

### Co-Immunoprecipitation Assay

For co-immunoprecipitation experiments, A549 cells (1 × 10^6^) were transfected with Flag-TRAF3 plasmid (2 μg/dish) and HA-UB (2 μg/dish) plasmids for 24 h. Then the transfected cells were infected with influenza virus WSN strain (MOI of 1) for 1 h. After infection, the cells were continued to be cultured with medium containing the Smac mimic AT406 (50 μM) to 20 h. At 4 h before sample collection, MG132 (10 μM) was added to prevent protein degradation by ubiquitination.

The cells were lysed with 500 µl IP buffer containing protease inhibitor and N-ethylmaleimide for 15 min. The protein supernatant was added into a Protein G Plus/Protein Agarose suspension-conjugated anti-Flag antibody after centrifugation at 14,000 rpm for 20 min. The cell lysate–bead mixture was then incubated at 4°C with rotation overnight. After incubation, the sample was centrifuged at 1,000 g for 1 min and carefully washed 3 times with IP Buffer. The remaining protein–bead mixtures were boiled in 50 µl 2× SDS loading buffer for 15 min and immunoblotted with anti-ubiquitin (Ub), TRAF3 antibodies.

### Plaque Assay

The TRAF3 knockdown or overexpression cells were infected with influenza virus WSN at MOI of 0.1. The cultural supernatants containing influenza progeny virus were harvested at 12 or 24 h postinfection (indicated in figure legends) and then applied for plaque formation assay to evaluate the virus titers. The overlay solution of plaque assay was prepared as described by Sang (Sang et al., 2021). Briefly, the solution containing 1 μg/ml TPCK-trypsin (Sigma-Aldrich, St. Louis, MO, USA) and 1% penicillin/streptomycin liquid (Gibco, Grand Island, NY, USA) was prepared by mixing 2× DMEM medium into an equivalent microcrystalline cellulose solution. After removing the virus inoculums, the virus-infected MDCK cells were re-covered with 3 ml overlay solutions per well. After culturing for an additional 72 h, the overlay was removed. The residue was washed up by PBS, and the live cells were fixed stained with crystal violet in 4% paraformaldehyde to observe the formation of plaques.

### Dual-Luciferase Reporter Assay

In mini-replicon assay (Yuan et al., 2015), 50 ng TRAF3 or vector control plasmid was co-transfected into 293T cells with 50 ng pPolI-Fluc, four plasmids comprising influenza virus vRNPs including pHW2K-PB1, pHW2K-PB2, pHW2K-NP, and pHW2K-PA plasmids, and 10 ng hRluc-TK plasmid using EndoFectin Transfection Reagent according to the instruction. After 8 h of transfection, the supernatants were removed and replaced by a fresh complete medium. 24 h later, the luciferase expression levels were detected by the Dual-Luciferase Reporter Assay system (Promega, USA).

In the study testing the effect of TRAF3 on IFN-β promoter activity, TRAF3 or control siRNA, IFN-β promoter plasmid with a luciferase reporter gene, and hRluc-TK internal reference plasmid were co-transfected into 293T cells. After transfection, the cells were treated and the luciferase values were detected as described above.

### ELISA

The expression IFN-β and cytokine in cultured supernatants were measured using commercial ELISA kits (Ruixin, Quanzhou, Fujian Province, China) according to the instructions of the manufacturer.

### Statistical Analysis

The results were expressed as mean ± SD of three independent experiments. Comparison between the two groups was performed by unpaired two-tailed Student’s *t*-test. One-way ANOVA was performed for overall differences (n > 3). The figures were analyzed by GraphPad Prism 7 software. The criterion for statistical significance was *p* < 0.05 and marked as an asterisk *, ** indicated *p* < 0.01, *** indicated *p* < 0.001.

## Results

### TRAF3 Can Regulate the Replication of IAV

**Figure 1:**
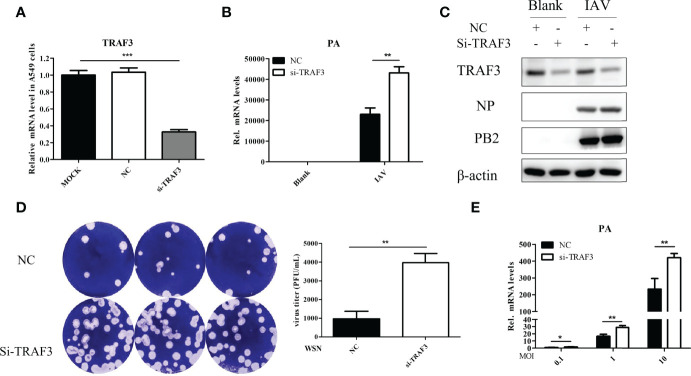
TRAF3 knockdown promotes influenza A virus infection. **(A)** A549 cells were transfected with TRAF3 siRNA or negative control (NC) siRNA for 24 h, and then the knockdown efficiency was detected by qRT-PCR (*p* < 0.001, vs. MOCK group). NC, negative control. **(B)** The siRNA-transfected A549 cells were infected with A/WSN/1933 (WSN, H1N1) for 24 h Then the effect of TRAF3 knockdown on influenza PA mRNA expression was detected by qRT-PCR. **(C)** Effect of TRAF3 knockdown on influenza NP and PB2 protein expression at 24 h postinfection were assessed by Western blotting. **(D, E)** The cultural supernatants containing influenza progeny virus were harvested at 24 h postinfection and then applied to plaque formation assay **(D)** and qRT-PCR **(E)** to verify the effect of TRAF3 knockdown on progeny virus production. Data were shown as mean ± SD (*p < 0.05, **p < 0.01, ***p < 0.001).

When the influenza virus binds to the host cell, its life cycle begins. Viral RNA is transcribed to synthesize viral mRNA after replication, then viral proteins are translated and assembled into newborn virions that are released outside the cell. To examine whether TRAF3 affects IAV replication, we performed a TRAF3 knockdown experiment using the siRNA system. A549 cells were transfected with siRNA targeting TRAF3, which led to about 70% knockdown of the target gene (**Figure 1A**). The qRT-PCR result indicated that the mRNA expression level of the influenza polymerase acidic (PA) subunit was significantly increased after A/WSN/1933 (WSN, H1N1) infection compared with that of the negative control (NC) group (**Figure 1B**). Meanwhile, the protein expression of influenza nucleoprotein (NP) and polymerase basic protein 2 (PB2) subunits with TRAF3 siRNA treatment were apparently higher than that of the control group (**Figure 1C**). Thus, our results indicated that knockdown of TRAF3 in A549 cells may promote the intracellular infection of influenza virus.

Once influenza virus has completed a replication cycle, it will be released from host cells. Plaque formation assay and qRT-PCR were used to detect the production of progeny virus in the cell supernatant after IAV infection with or without TRAF3 knockdown. The results demonstrated that the number of plaque formations (**Figure 1D**) and viral PA mRNA expression levels (**Figure 1E**) in the TRAF3 knockdown group were significantly higher than those in the NC group, indicating that TRAF3 knockdown would increase the production of influenza progeny virions. It is important to highlight that TRAF3 knockdown could influence the ability of the host to overcome influenza virus infection.

**Figure 2:**
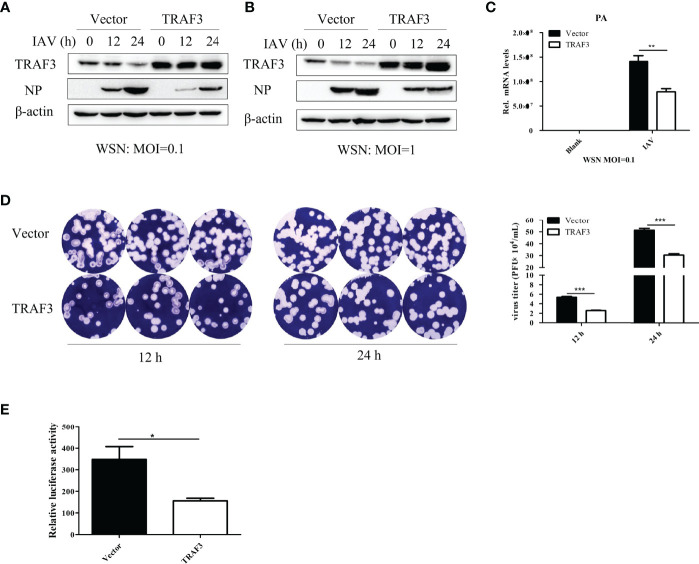
Overexpression of TRAF3 inhibits influenza A virus infection. **(A, B)** TRAF3-overexpressing or vector control stable cell lines were infected with WSN virus under the treatment of different virus titers (MOI = 0.1 or 1) and infection time (12 or 24 h). The influenza NP protein expression was measured by Western blotting. **(C)** Effects of TRAF3 overexpression on influenza PA mRNA expression was tested by qRT-PCR at 24 h postinfection. **(D)** The cell supernatants were collected in TRAF3 overexpression or control groups at 12 and 24 h postinfection at MOI of 0.1, then the virus titer was assessed by plaques formation assay, and the statistical analysis of it was measured. **(E)** TRAF3 or vector control plasmid was co-transfected with influenza vRNP plasmids into 293T cells, and the vRNP activity was detected by mini-replicon assay. Data were shown as mean ± SD (*p < 0.05, **p < 0.01, ***p < 0.001).

To further examine the effect of TRAF3 on the replication of IAV, the TRAF3 overexpression and vector control stable cell lines were constructed by A549 cells as described in *Material and Method*, and the cells were infected with IAV at different infection titers (MOI = 0.1 or 1). The cells were harvested at 12 or 24 h postinfection and then analyzed by qRT-PCR and Western blotting. The supernatants were collected for plaque assay to determine the virus titers. As shown in **Figures 2A, B**, compared with those in control cells, the expression levels of influenza virus NP protein were found to be reduced in TRAF3-overexpressing cells. The same trend was observed in the expression level of influenza PA mRNA (**Figure 2C**). Furthermore, the plaque formation of the tested viruses in the TRAF3 overexpression group was markedly reduced compared with the vector control group (**Figure 2D**), indicating that the overexpression of TRAF3 might contribute to the reduction of progeny virus production. Based on the results above, we demonstrated that overexpression of TRAF3 could impair IAV replication, while knockdown of TRAF3 has the opposite effect. These phenomena demonstrate that TRAF3 might be a factor in restricting the replication of IAV.

The viral ribonucleoprotein complexes (vRNPs) are independent functional units which mediated the transcription and replication of the viral genome. To determine whether TRAF3 is involved in the regulation of influenza virus polymerase activity, we performed mini-replicon assay to elevate polymerase activity. As shown in **Figure 2E**, the fluorescence value of the system was significantly decreased in the TRAF3 group upon IAV infection, indicating that influenza virus polymerase activity is regulated by TRAF3. The result further confirmed that the presence of TRAF3 could facilitate the reduction of influenza virus titer. Since the signaling adaptor TRAF3 is a regulator of innate immunity, we then explored the role of TRAF3 in the innate immune signaling pathway triggered by IAV infection.

### TRAF3 Promotes IRF3-Initiated Type I IFN Production in the Innate Response Against IAV Infection

**Figure 3:**
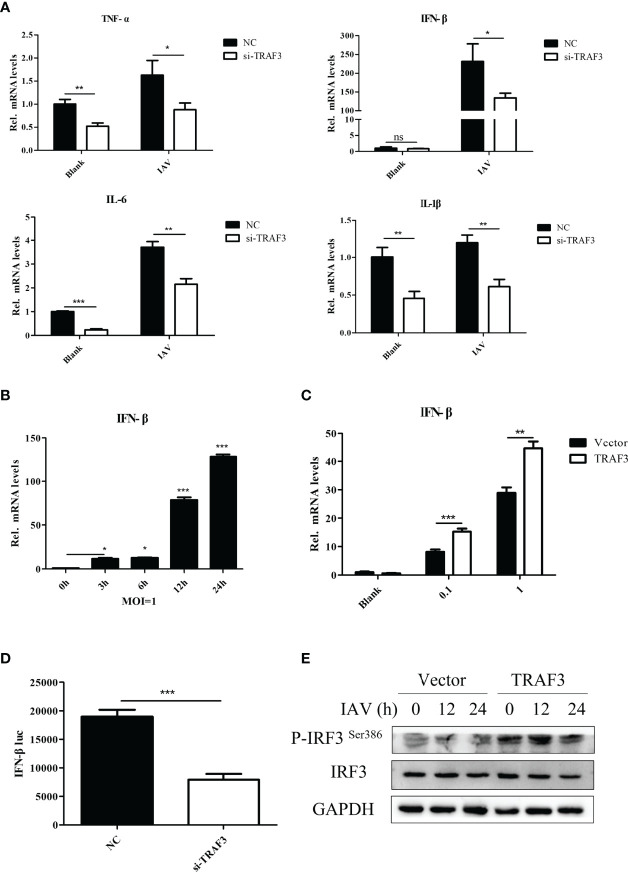
The regulation of TRAF3 on IAV replication is related to the type I IFN signaling pathway. **(A)** The siRNA-transfected cells were infected with WSN virus at MOI of 1 for 24 h, then the proinflammatory cytokine mRNA expressions were detected by qRT-PCR. **(B)** Influenza-induced IFN-β mRNA expression with the time of infection at MOI of 1 was detected by qRT-PCR (*p < 0.05, ***p < 0.001, vs. 0-h group). **(C)** The influenza virus induced IFN-β mRNA expression influenced by TRAF3 overexpression at different virus titers were measured by qRT-PCR at 24 h postinfection. **(D)** NC/TRAF3 siRNA were co-transfected with the IFN-β promoter plasmid carrying luciferase reporter gene and hRluc-TK internal reference plasmid into 293T cells for 24 h The IFN-β promoter activity was verified by dual luciferase reporter assay. **(E)** The TRAF3 overexpression cells were infected with WSN virus at MOI of 1 for 24 h; the phosphorylation of IRF3 Ser386 was detected by Western blotting. Data were shown as mean ± SD (**p < 0.01, ***p < 0.001). “ns” stands for no significance, ns p>0.05.

Antiviral immune response is well known to impair viral infection. Increasing evidence indicated the crucial contributions of cytokines in the innate immune response to intracellular pathogens. After knockdown of TRAF3, we detected the production of inflammatory cytokines in WSN-infected A549 cells by qRT-PCR. It was found that the silence of TRAF3 resulted in the decrease in pro-inflammatory cytokines such as TNF-α, IFN-β, IL-6, and IL-1β (**Figure 3A**). Our results revealed the importance of TRAF3 in mediating immune reactions during IAV infection.

Since TRAF3 is an essential adaptor of innate immunity to produce type I IFN in response to IAV infection (Lin et al., 1998), we identified the signaling pathway in which TRAF3 is involved. Expression analysis showed that IFN-β could be activated upon IAV stimulation (**Figure 3B**). Notably, overexpression of TRAF3 was shown to markedly enhance the expression of IFN-β mRNA (**Figure 3C**) and protein (**Figure S1**) levels induced by influenza virus infection in comparison with the control group, which is contrary to the results of TRAF3 knockdown shown in **Figure 3A**. Hence, signaling pathway analysis revealed that the upregulation of the type I IFN signaling pathway was associated with TRAF3.

Next, we investigated whether the immunoregulatory capacity of TRAF3 was associated with the transcriptional activity of the IFN-β promoter. No-target siRNA or siRNA against TRAF3 was co-transfected into 293T cells with the IFN-β promoter plasmid carrying the luciferase reporter gene and internal reference plasmid for dual-luciferase assay. We found that the IFN-β promoter activity in the TRAF3 knockdown group was remarkably decreased compared with the NC group (**Figure 3D**), indicating that TRAF3 could regulate the expression of IFN-β by affecting the activity of the IFN-β promoter.

Previous studies reported that viral infection could induce the phosphorylation and dimerization of interferon regulatory factor 3 (IRF3), an important transcription factor in innate antiviral immunity, followed by triggering of the transcription and expression of type I IFNs (Mori et al., 2004; Tsuchida et al., 2009). We wondered whether the regulator effect of TRAF3 on IAV replication was positively associated with the activation of IRF3. Upon IAV infection, the level of phosphorylation of IRF3 Ser386 in TRAF3-overexpressing cells was detected by Western blotting. Compared with the vector control, the phosphorylation of IRF3 was significantly increased in TRAF3-overexpressing cells (**Figure 3E**), suggesting that TRAF3 could promote IRF3-induced IFN-β promoter activity. Importantly, the phosphorylation of IRF3 and activation of specific promoters activated the IFN system (Schneider et al., 2014), leading to the transcription of numerous interferon-stimulated genes (ISGs). In our experiment, we observed that IAV-induced ISG expression could be promoted by the overexpression of TRAF3 in A549 cells, including IAIG15, OAS1, IFI6, RSAD2, and MX1 (**Figure S2**). These data indicated that TRAF3 promoted IRF3-initiated antiviral innate immunity, resulting in a positive effect on anti-influenza virus infection.

### Inhibiting TRAF3 Degradation Due to IAV Infection Is Correlated With Host Anti-Influenza Innate Immunity

**Figure 4:**
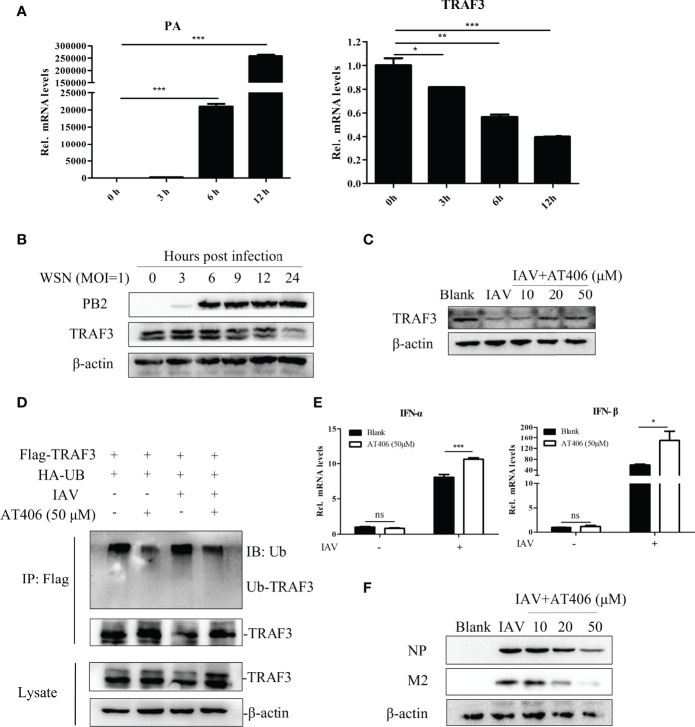
Inhibiting TRAF3 protein degradation correlates with the regulatory mechanism of antiviral immunity. **(A)** The mRNA expression of influenza PA and TRAF3 at different time points of infection were detected by qRT-PCR (*p < 0.05, **p < 0.01, ***p < 0.001, vs. 0-h group). **(B)** The protein expression of PB2 and TRAF3 under IAV infection at MOI of 1 were detected by Western blotting. **(C)** The Smac mimic AT406 was added into A549 cells after the tested virus infection (MOI = 1) and cultured to 24 h, then the TRAF3 protein expression was identified by Western blotting. **(D)** Effects of Smac mimic AT406 on IAV-induced ubiquitination of endogenous TRAF3. A549 cells were co-transfected with the HA-Ub and Flag-TRAF3 plasmids (2 μg/dish). At 24 h after transfection, the transfected cells were infected with IAV (MOI of 1) for 1 h and then cultured in mediums containing AT406 (50 μM). At 4 h before sample collection, MG132 (10 μM) was added to prevent protein degradation by ubiquitination. The cell lysates were immunoprecipitated with anti-Flag. The immunoprecipitates were analyzed by immunoblots with anti-ubiquitin and anti-TRAF3 as indicated. **(E)** The mRNA expression of type-I IFNs under AT406 treatment after the tested virus infection (MOI = 1) was measured by qRT-PCR 24 h postinfection (*p < 0.05, ***p < 0.001). **(F)** The influenza NP and M2 protein expression under AT406 treatment after the tested virus infection (MOI = 1) was verified by Western blotting 24 h postinfection. “ns” stands for no significance, ns p>0.05.

To determine the changes of TRAF3 in response to IAV stimulation, we simultaneously detected the expression of influenza PA and TRAF3 mRNA levels in the cells infected with IAV. With the virus infection, the expression level of influenza PA mRNA gradually increased, but the expression of TRAF3 mRNA gradually decreased (**Figure 4A**). The same trend was observed for influenza PB2 and TRAF3 protein levels (**Figure 4B**). Our results indicated that IAV infection diminished the TRAF3 expression at both mRNA level and protein level. The reduced TRAF3 expression might be crucial for virus replication, which intrigued our interest to explore the regulatory mechanism for TRAF3 under IAV infection.

As for the protein level, the regulatory system of intracellular protein degradation can be roughly divided into autophagy-lysosome system and Ub-proteasome system (Jang, 2018). We wonder whether TRAF3 degradation induced by IAV infection is undertaken by these two classical pathways. We infected the cell with IAV in the presence of the autophagy lysosome inhibitor bafilomycin at the concentration of 20 nM for 12 or 4 h and checked the TRAF3 expression by Western blot. The result showed that bafilomycin could not prevent the IAV-induced degradation of TRAF3 (**Figure S3**), suggesting that the degradation of TRAF3 induced by IAV infection was independent of the autophagy-lysosomal degradation pathway.

Further, does the IAV-induced degradation of TRAF3 depend on the well-characterized ubiquitin proteasome system? As reported, the inhibitor of apoptosis proteins (cIAPs) is the component of the TNF receptor complex and can interact with the TRAF family to regulate downstream signal transduction as a ubiquitin ligase (Dougan and Dougan, 2018). Also, it has been shown that cIAP1/2 mediated the virus-triggered ubiquitination of TRAF3, which is essential for the induction of type I IFN and cellular antiviral response (Mao et al., 2010). Moreover, the degradation of TRAF3 could be blocked by small-molecule cIAP antagonists like Smac (Xiao et al., 2013). Thus, we hypothesized that IAV-induced TRAF3 degradation might be related to cIAP1/2. The Smac mimic AT406 (SM406) is a novel and orally active antagonist of multiple IAP proteins (Cai et al., 2011). We treated IAV-infected cells with cIAP antagonist mimic AT406 and tested TRAF3 degradation by Western blot. As shown in **Figure 4C**, TRAF3 degradation was significantly blocked in the presence of the Smac mimic AT406 in IAV-infected A549 cells, indicating that virus-triggered TRAF3 degradation could be inhibited by the cIAP antagonist. To further confirm whether the TRAF3 degradation disturbed by the cIAP antagonist is dependent on IAV-induced TRAF3 ubiquitination, we performed co-immunoprecipitation (Co-IP) assay. As shown in **Figure 4D**, the TRAF3-linked ubiquitination in the IAV-treated group was obviously increase compared with the non-infection group. Meanwhile, AT406 treatment significantly inhibited IAV-induced ubiquitination of TRAF3. Taken together, the observed degradation of TRAF3 induced by IAV infection is associated with cIAP-mediated ubiquitination.

The degradation of TRAF3 showed to be important for association with the production of IFNs (Kayagaki et al., 2007). Since AT406 abrogated cIAPs’ ability to interact with TRAF3, we then analyze the induction of type I IFN. The results displayed that the expression levels of type I IFN (IFN-α and IFN-β) mRNA (**Figure 4E**) and IFN-β protein (**Figure S4**) expression levels were significantly increased after treatment of AT406. Meanwhile, influenza virus protein levels decreased gradually with the increase in the concentration of AT406 (**Figure 4F**). Thus, the result clearly showed that inhibiting the cIAP-triggered ubiquitination of TRAF3 induced by the tested virus was beneficial for IFN-β production, which consequently induced innate immune responses. More importantly, maintaining the stable expression level of TRAF3 may contribute to its regulatory role in the innate immune system in defending against virus infection.

### The Zinc Finger, Coiled-Coil, and MATH Domains of TRAF3 Are Essential for Positively Regulation of the IFN Antiviral Response

**Figure 5:**
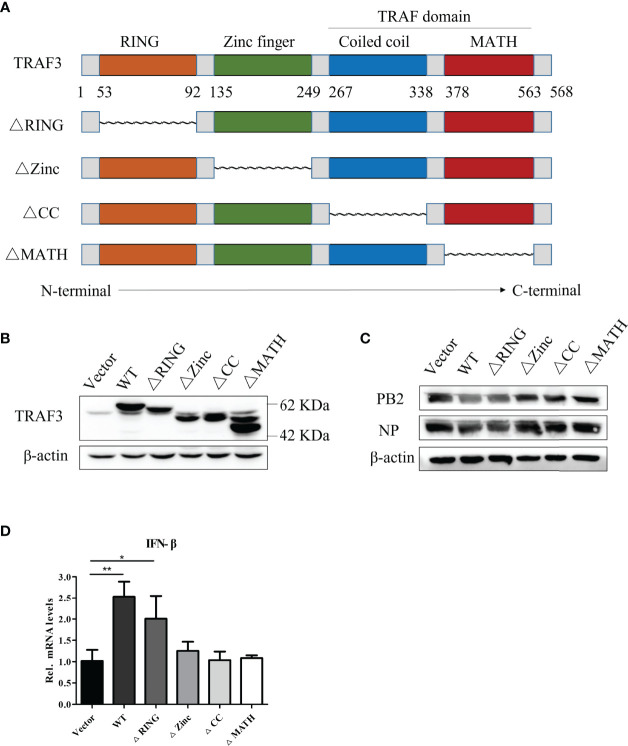
Anti-influenza A virus effect of different structural domains of TRAF3. **(A)** Structural simulation of TRAF3 domains, data from NCBI (NM_145725.3, NP_663777.1) and UniProt (UniProt ID: Q13114). **(B)** Protein expression of domain deletion isoforms in full-length TRAF3 were detected by Western blotting. **(C)** The stable expression cell lines with domain deletion isoforms were infected with WSN virus at MOI of 1 for 24 h, then the influenza PB2 and NP protein expressions were analyzed by Western blotting. **(D)** The regulatory effects of TRAF3 domain deletion isoforms on the virus-induced (MOI = 1) IFN-β mRNA expression were determined by qRT-PCR at 24 h post virus infection. (*p < 0.05, **p < 0.01).

As described in *Introduction*, the TRAF family has a similar secondary structure and can be divided into different domains, including an N-terminal RING, two zinc finger, coiled-coil (CC), and C-terminal MATH domains. Here, we constructed stable expression cell lines with deletions in the RING, zinc finger, coiled-coil, and MATH domains to compare the potential regulatory effect of different domains of TRAF3 in IAV-infected A549 cells. The structural simulation of TRAF3 domain deletions is shown in **Figure 5A**. The TRAF3 monoclonal antibody, which recognizes the amino acid residues near TRAF3 Gly38, could be used to detect the expression of TRAF3 protein with domain deletion to verify whether the deletions were successfully constructed. As shown in **Figure 5B**, among the stable expression cells with deletion of each domain, the protein length of TRAF3 ranged from 42 to 62 kDa, which was consistent with the length of the gene after each deletion. Moreover, the expression of the protein levels was greatly increased compared with the vector control group, indicating that the stable expression cells of TRAF3 deletions with each domain were successfully constructed.

To detect the effect of the deletion of each domain on the expression of influenza virus proteins, we infected each group of cells with the WSN virus at MOI of 1 and measured the expression of influenza PB2 and NP proteins at 24 h postinfection. As shown in **Figure 5C**, the TRAF3-WT group significantly reduced the expression of influenza PB2 and NP proteins compared with the vector group, which was consistent with the previous results. In the cells with different-domain deletion, RING finger domain deletion still displayed the anti-IAV effect, which was equivalent to that of the WT group, indicating that the partial deletion of the RING domain in TRAF3 has no impact on the regulator stability. However, the deletion of the remaining domains, including the zinc finger, coiled-coil, and MATH domains, failed to restore the antiviral response, suggesting that these three domains participated in the positive regulation of IAV replication.

As described above, we have proved that TRAF3 interfered with viral replication *via* activating the type I IFN signaling pathway. Hereby, we continue to detect the impact of TRAF3 domain deletion on IFN antiviral response after IAV infection. It was found that the group-deleted RING finger domain significantly enhanced the intracellular IFN-β mRNA expression compared with the control group (**Figure 5D**), which was similar to the TRAF3-WT group, whereas the mRNA expression of type I IFN showed no statistical significance between the control group and the groups of the deleted zinc finger, coiled-coil, and MATH domains, respectively. Therefore, we inferred that the zinc finger, coiled-coil, and MATH domains were required for the TRAF3 activation of IFN antiviral response genes.

## Discussion

The rapid immune response to virus infection can produce a broad-spectrum virus-killing effect and specific antibodies. Further study on the antiviral effect of host immune regulators has great potential in the face of influenza pandemics with new and unknown strains.

Members of the TRAF family are mainly involved in the regulation of inflammation, antiviral response, and apoptosis. Among them, TRAF3 is an important effector of innate immunity against viral infections by inducing antiviral response. Recruiting TRAF3 to MAVS is an important step of RIG-I-MAVS antiviral signal transduction (Paz et al., 2011; Zhu et al., 2019). In previous studies, TRAF3 had been found to have a positive impact on VSV (Oganesyan et al., 2006), HPV (Zhang et al., 2018), HSV (Perez et al., 2010), etc., and some related diseases. However, the effect of TRAF3 on anti-influenza A virus infection and its mechanism remain unclear and worth exploring in depth.

The effects of the knockdown and overexpression of TRAF3 on virus infection were evaluated. At the beginning, we demonstrated that TRAF3 exhibits a positive function in restraining virus propagation. Furthermore, the activity of influenza virus mini-replicon vRNPs was also significantly inhibited in TRAF3 overexpression cells, suggesting that TRAF3 was downregulated upon infection with influenza virus by affecting the activity of vRNPs. The results of our study are consistent with the previously published study (Sun et al., 2020). vRNPs consist of influenza PB2, PB1, PA, and nucleoprotein (NP), together with viral RNA, which is the functional unit for the transcription and replication of the viral genome. It has been suggested that both viral PB2 and polymerase basic protein 1-frame 2 (PB1-F2) associate with MAVS to suppress the production of type I IFN (Graef et al., 2010; Varga et al., 2011). Based on these results, we infer that TRAF3 plays a positive role in the host innate immune response to influenza virus infection.

Type I IFNs (α and β) induce the synthesis of a variety of IFN-stimulated genes (ISGs) to help the host present the antiviral state and establish innate immune responses to defend against viral infection (Paz et al., 2011). In this process, IRF3 plays an important role in the production of type I IFNs induced by virus infection (Tsuchida et al., 2009). In order to escape the host’s innate immune response, many viruses have acquired mechanisms that suppress IRF3 activation to escape from type I IFN effects (Chan and Gack, 2016; Schulz and Mossman, 2016). In this regard, regulating IRF3 activation has become the target of therapeutic strategies for viral infections and autoimmune diseases. TRAF3 is a major regulator of innate antiviral response, which is essential for type I IFN production (Hacker et al., 2006; Oganesyan et al., 2006). Specifically, TRAF3 can stimulate IKK-related kinases, TBK1 and IKKϵ, thereby inducing phosphorylation of the C-terminal of IRF3/IRF7, leading to IRF3 dimerization and nuclear translocation, DNA binding, and IRF-dependent antiviral gene activation (Sharma et al., 2003). Therefore, the activation of type I IFNs can be evaluated by detecting the phosphorylation of IRF3. We found that knocking down TRAF3 can effectively reduce the mRNA and protein expression of pro-inflammatory factors like TNF-α, IL-6, IL-1β, and IFN-β induced by influenza virus infection. In addition, the regulation of TRAF3 on IFN-β was related to its impact on the activity of the IFN-β promoter. The phosphorylation level of IRF3 Ser386 was also increased by TRAF3 overexpression, which reflects the activation of type I IFN signaling. Consistently, the increase in ISG expressions also confirmed the activating effect of type I IFNs. These results imply a favorable role of TRAF3 in the activation of the type I IFN signaling pathway and the production of the antiviral factors and ISGs induced by influenza virus infection, which may suggest a potential therapeutic strategy for IAV infection.

Accumulating evidence suggested that TRAF3 can be degraded through ubiquitination and activation of cIAP1/2, which is essential for type I IFN induction and cellular antiviral response (Mao et al., 2010; Lin et al., 2015). The degradation of TRAF3 stimulated by macrophage colony-stimulating factor (M-CSF) was blocked by Smac, the small-molecule cIAP antagonist (Jin et al., 2014). In our research, we have found that TRAF3 degradation triggered by influenza virus infection was not dependent on the autophagy lysosomal system. Notably, IAV-induced TRAF3 ubiquitination could be blocked by the cIAP1/2 antagonist AT406. Influenza virus-induced TRAF3 degradation was inhibited by cIAP1/2 antagonist AT406, which could also decrease the protein expression of influenza virus and promote IFN production. Based on these results, we concluded that maintaining the stable expression of TRAF3 protein after influenza virus infection was beneficial to host cells to produce antiviral factors, thereby preventing the potential immune escape of influenza virus by terminating type I IFN response.

Based on the significant effect of TRAF3 on anti-influenza virus infection and type I IFN signaling pathway, we further explored the anti-influenza virus effect of each domain of TRAF3 by constructing mutants with deletion of each domain. TRAF3 is defined by the presence of a RING finger domain, five zinc finger domains, and a TRAF domain. Our study would suggest that the zinc finger domain and TRAF domain (including coiled-coil domain and MATH domain) in full-length TRAF3 were essential to fighting against IAV infection. Conversely, the RING domain was a non-essential domain in antiviral infection. Evidence has accumulated to support that both homo- and heterodimers mediated by the TRAF RING domains can synthesize ubiquitin chains (Budhidarmo et al., 2012; Buetow and Huang, 2016; Middleton et al., 2017). Moreover, many TNF superfamily receptors have a TRAF-interacting motif and depend on TRAFs for signal transduction. For example, the TRAF6-TRAF2 and TRAF6-TRAF5 RING heterodimers assemble ubiquitin chains (Das et al., 2021). We infer that the above domains of TRAF3 might be related to inhibition of the degradation of TRAF3, while the RING domain of TRAF3 might be responsible for building ubiquitin chains. Thus, we have emphasized that the zinc finger, coiled-coil, and MATH domains of TRAF3 are essential for the positive regulation of IAV replication, and their possible connection with pathogenesis. However, the exact molecular mechanisms of the three domains in the formation of TRAF3 networks, as well as their impact on antiviral effects, are not well understood. Based on these findings, we will explore the functional characterization of the three domains of TRAF3 in response to virus infection in our future work. We assume that the three domains form a platform which facilitates activation of downstream kinases.

**Figure 6:**
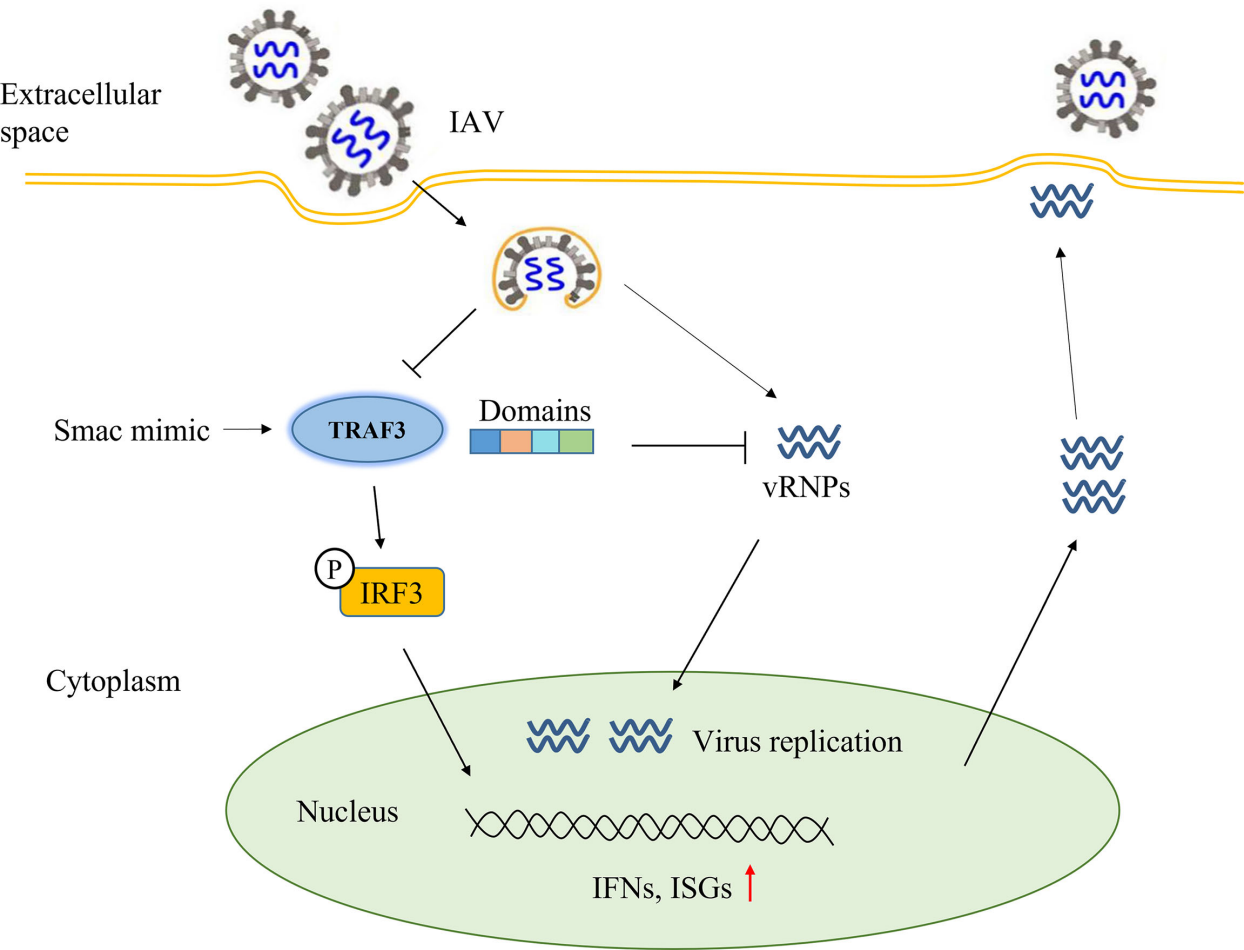
Summary diagram of full text: the regulatory effect of TRAF3 on influenza virus replication.

In conclusion (**Figure 6**), our study demonstrated that TRAF3 could affect IAV replication and promote the expression of IFN-β and ISGs detected in the infected cells. Maintaining the stable expression of TRAF3 and avoiding its ubiquitinated degradation triggered by influenza virus would facilitate the production of type I IFNs, which could reduce the immune evasion of influenza viruses and help in virus clearance. Among the full-length TRAF3, both the ZING finger domain and TRAF domain are necessary for TRAF3 to participate in the regulation of IAV replication. Our study revealed that TRAF3 as a positive regulator is involved in the antiviral defense against IAVs and validates TRAF3 as a potential therapeutic for IAV infection.

## Data Availability Statement

The original contributions presented in the study are included in the article/**Supplementary Material**. Further inquiries can be directed to the corresponding author.

## Author Contributions

All authors listed have made a substantial, direct and intellectual contribution to the work, and approved it for publication.

## Funding

This work was supported by the National Natural Science Foundation of China (Grant number 82073897), Guangdong Basic and Applied Basic Research Foundation (Grant number 2019A1515011550), and Construction Project of Influenza Prevention and Treatment Technology System of Chinese Medicine organized by Chinese Academy of Traditional Chinese Medicine (Grant number ZZ13-035-02).

## Conflict of Interest

The authors declare that the research was conducted in the absence of any commercial or financial relationships that could be construed as a potential conflict of interest.

## Publisher’s Note

All claims expressed in this article are solely those of the authors and do not necessarily represent those of their affiliated organizations, or those of the publisher, the editors and the reviewers. Any product that may be evaluated in this article, or claim that may be made by its manufacturer, is not guaranteed or endorsed by the publisher.
