# Supplementary material for: TRAF3 Positively Regulates Host Innate Immune Resistance to Influenza A Virus Infection
Source: Front Cell Infect Microbiol. 2022 Apr 27;12:839625. doi: 10.3389/fcimb.2022.839625 (PMC9093644; doi:10.3389/fcimb.2022.839625)
Supplement: Supplementary file 1 [file DataSheet_1.docx]

**Supplementary material**

**TRAF3 positively regulates host innate immune resistance to influenza A virus infection**

**Fangzhao Chen^1,2#^, Liurong Chen^1#^, Yinyan Li^1^, Huiting sang^1^, Chunyu Zhang^1^, Shuofeng Yuan ^3,4^, Jie Yang^1,*^**

^1^ NMPA Key Laboratory for Research and Evaluation of Drug Metabolism, Guangdong Provincial Key Laboratory of New Drug Screening, School of Pharmaceutical Sciences, Southern Medical University, Guangzhou 510515, China

^2^ Shenzhen Children's Hospital, Shenzhen 518048，China

^3^ Department of Microbiology, Li Ka Shing Faculty of Medicine, The University of Hong Kong, Pokfulam, Hong Kong SAR, China.

^4^ State Key Laboratory of Emerging Infectious Diseases, Li Ka Shing Faculty of Medicine, The University of Hong Kong, Pokfulam, Hong Kong SAR, China.

*** Correspondence:**Corresponding Author
E-mail address: yj528@smu.edu.cn (Jie. Y)

^#^These authors contributed equally.


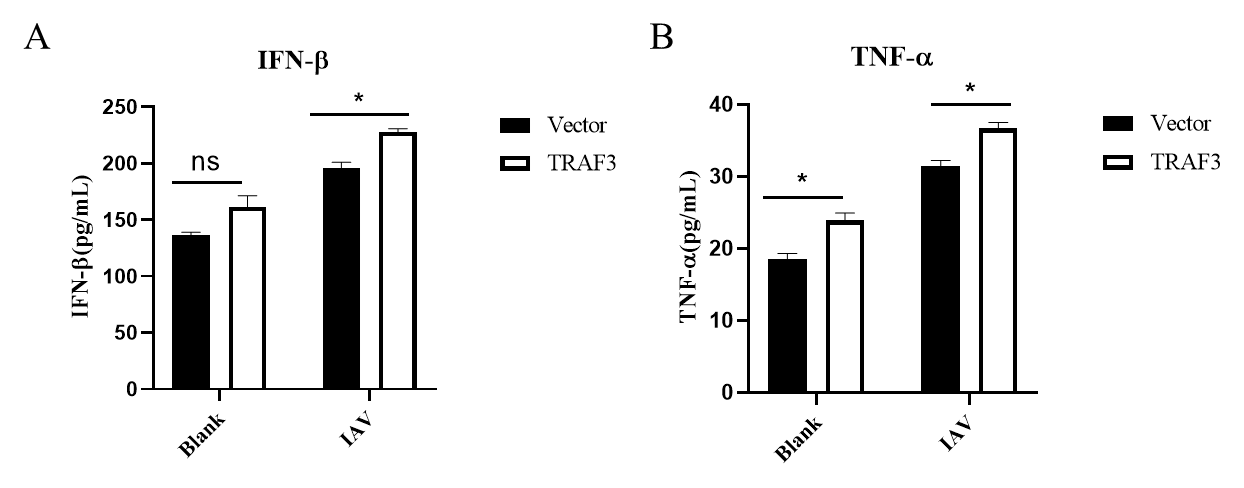


Fig. S1. The protein expression of IFN-β (A) and TNF-α (B) in TRAF3 overexpressing or vector control stable cell line was measured by ELISA at 24 h post infection (*p < 0.05).


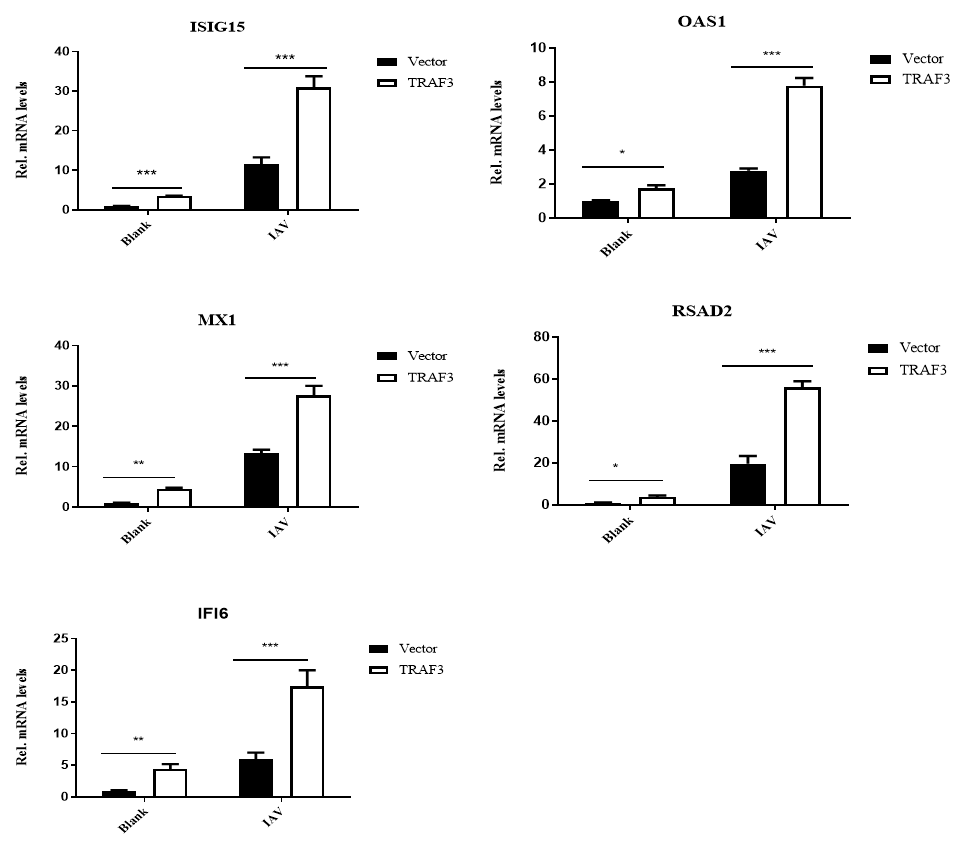


Fig. S2. The TRAF3 overexpression cells were infected with WSN virus at MOI of 1 for 24 hours, then mRNA levels of ISGs were detected by qRT-PCR. Data were shown as mean ± SD (**p* < 0.05, ***p* < 0.01, ****p* < 0.001)


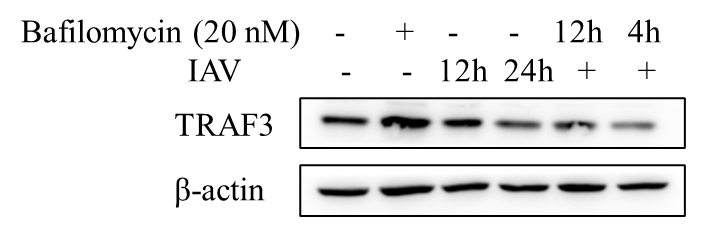


Fig. S3. After the virus infection (MOI=1), the autophagy lysosome inhibitor Bafilomycin (20 nM) was added into the infected cells at 12 h and 4 h before sample collection at 24 h post infection, the expression of TRAF3 protein was analyzed by Western blotting.


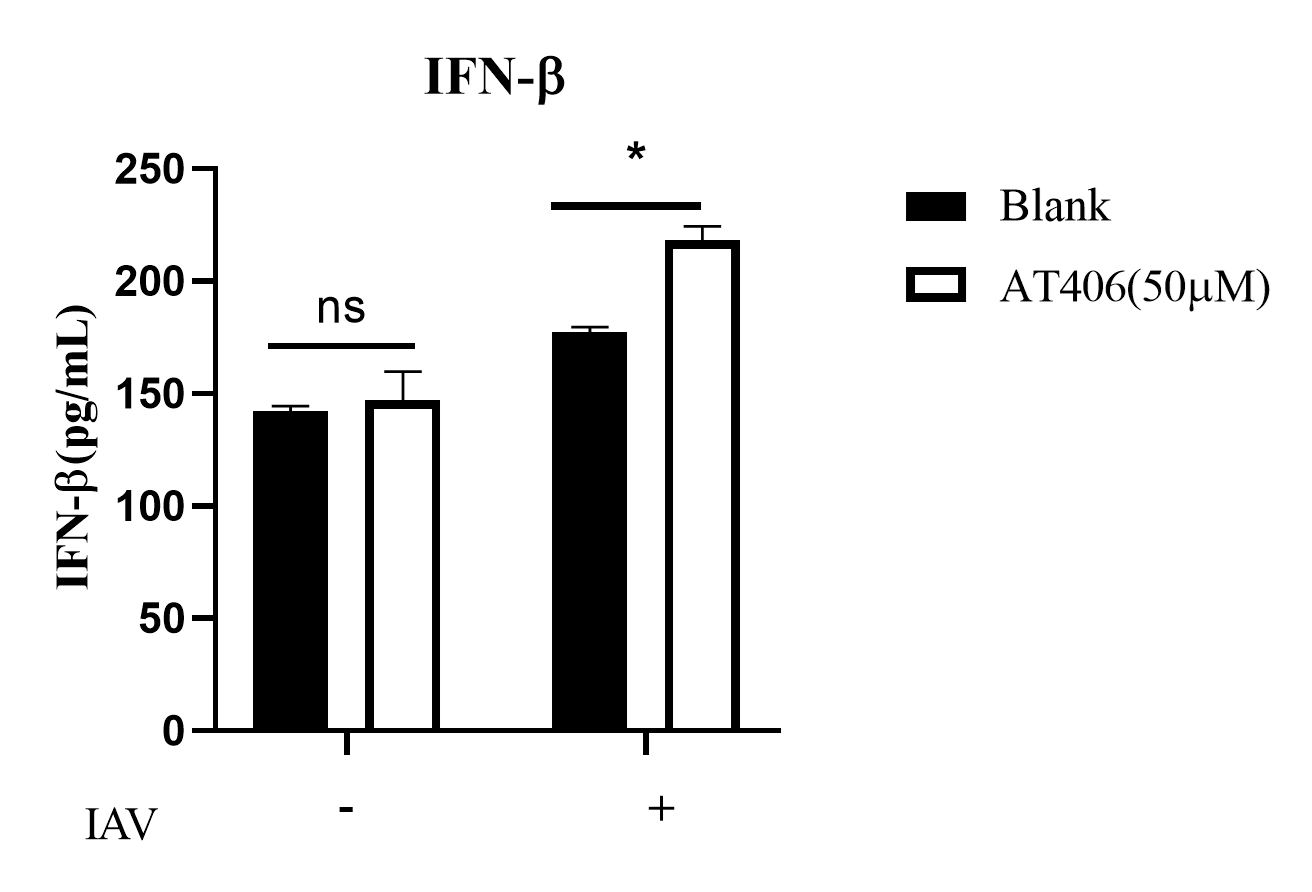


Fig. S4. The protein expression of IFN-β under AT406 (50 μM) treatment after WSN virus infection (MOI=1) was measured by ELISA at 24 h post infection (*p < 0.05).

Table S1. Primer sequences for qRT-PCR

| Name | Primer sequences |
| --- | --- |
| IFI6-Forward | GGTCTGCGATCCTGAATGGG |
| IFI6-Reverse | TCACTATCGAGATACTTGTGGGT |
| ISG15-Forward | CGCAGATCACCCAGAAGATCG |
| ISG15-Reverse | TTCGTCGCATTTGTCCACCA |
| OAS1-Forward | TGTCCAAGGTGGTAAAGGGTG |
| OAS1-Reverse | CCGGCGATTTAACTGATCCTG |
| MX1-Forward | GTTTCCGAAGTGGACATCGCA |
| MX1-Reverse | CTGCACAGGTTGTTCTCAGC |
| RSAD2-Forward | TGGGTGCTTACACCTGCTG |
| RSAD2-Reverse | GAAGTGATAGTTGACGCTGGTT |
